# Editorial: Polysubstance Abuse and Cognitive Dysfunction

**DOI:** 10.3389/fnbeh.2022.916921

**Published:** 2022-07-26

**Authors:** Bruno Kluwe-Schiavon, Thiago Wendt Viola, Rodrigo Grassi-Oliveira, Saulo Gantes Tractenberg

**Affiliations:** ^1^Developmental Cognitive Neuroscience Lab (DCNL), Brain Institute of Rio Grande Do Sul, Pontifical Catholic University of Rio Grande Do Sul (PUCRS), Porto Alegre, Brazil; ^2^Research Center for Psychological Science, University of Lisbon (ULisbon), Alameda da Universidade, Lisbon, Portugal; ^3^Translational Neuropsychiatry Unit, Department of Clinical Medicine, Aarhus University, Aarhus, Denmark

**Keywords:** addiction, substance use disorder, cognition, substance abuse, dependence

Substance use disorder (SUD) is a chronic-multistage psychiatric disease characterized by disturbances in different brain functional and anatomical levels (Koob and Volkow, 2016), associated with cognitive, behavioral, and social dysfunction. Specifically, cognitive dysfunction has been found as an important predictor of a worse prognosis over the course of SUD (Kroll et al., 2018; Bruijnen et al., 2019; Hirsiger et al., 2019). Yet, one important factor that has been widely described in clinical practice is the fact that a considerable large number of people with SUD will report a history of a complex pattern of polysubstance use (PSU), defined as using multiple substances within a specified period during their lives (Conway et al., 2013; John et al., 2018; Crummy et al., 2020). Hence, when trying to better understand the neurobehavioral mechanisms of the transition from the first impulsive use to the compulsive consumption pattern (Aguilar et al., 2020), this fact constitutes an additional methodological challenge for researchers in the addiction field, since it seems almost impossible to detach the main effect of one specific drug on cognitive dysfunction, or even to consider all the biopsychosocial factors that, in interaction with PSU, contribute to cognitive dysfunction and worsen prognosis. Addressing the desire for addiction research to bridge the gap between PSU trajectories of SUDs and the influence of biopsychosocial factors (Rounsaville et al., 2003; Lee et al., 2010), this editorial aims to explore the biopsychosocial factors that might relate to PSU and possibly aggravate the cognitive dysfunction and the course of SUD.

Aiming to investigate the possible individual and contextual factors that motivate initial substance consumption behaviors, Steinhoff et al. performed a community-representative, long-term longitudinal study using data collected from more than 5,000 individuals (ages 13–20) and found an increase in PSU between early adolescence to early adulthood. The authors elucidated important risk factors for PSU progression, including the exposure to others‘ substances, prenatal (e.g., maternal substance use during pregnancy), and childhood factors, as examples of childhood sensation-seeking (Steinhoff et al.). The PSU progression in early adulthood is alarming and its consequences on cognitive development need attention. In this regard, the study by Bourgault et al., also presented on this topic, assessed more than 36,000 adults, and showed that an additional SUD is associated with a decrease in self-reported cognitive performance. Interestingly, the authors found PSU women had a stronger decrease in attention when compared to men (Bourgault et al.), highlighting the discussion of potential sex differences in cognitive functioning among PSU. In the same line, another study on this special topic retrospectively accessed case files from 40 individuals with PSU who reported methamphetamine as their main drug of choice, and compared them with 27 individuals with alcohol use disorder–they found that some of the most frequent cognitive functioning outcomes are primarily driven by a vast range of biopsychosocial and neuropsychological individual factors (Gooden et al.). Specifically, the authors discussed that it is often not possible to attribute causality or identify methamphetamine use as a specific etiological factor for cognitive impairment, as many other demographic, neurodevelopmental, medical, psychiatric, or even PSU factors are well known to significantly influence the overall cognitive functioning. In line with that, a multilevel meta-regression study that included demographic and clinical moderators from 88 studies demonstrated that while remission might have a positive effect on learning through errors, the early onset of substance consumption and psychiatric comorbidities are associated with the lower valuation of delayed gratification, suggesting that alterations in the valuation of reward could precede deficits in error-driven learning during in SUDs (Kluwe-Schiavon et al., 2020). Curiously, contrary to these findings, one study included in this topic compared 54 substance abusers with 28 self-reported health controls and found only a trend of impulsiveness and risk-taking behaviors in substance abuse participants, although no statistically significant differences between the groups were found after a battery of decision-making tasks (Mejia et al.).

Not surprisingly, in clinical practice professionals are often requested to evaluate the presence of cognitive impairment as a result of PSU, precisely because there are concerns that severe drug consumption leads to alterations in brain structure and function (Gooden et al.). Thus, in light of the complexity of this biopsychosocial disorder, neuroimaging studies are also adapting toward broadly exploring brain connectivity instead of singular areas. Although it is known that SUD is associated with gray matter loss, particularly in the frontal cortex (Connolly et al., 2013), a study investigated if these regional gray matter alterations are independent of each other or the result of system-level processes at the intrinsic connectivity network level, and to what degree these regional gray matter alterations are substance-specific or shared across different substances (Muller et al.). With a sample composed of different SUD patients the study, which is also presented in this special topic, found that the default mode network was most affected in the alcohol use disorder, followed by the salience and executive control networks, whereas the salience and somatomotor network were highlighted as critical for understanding opioid use disorder (Muller et al.). Importantly, PSU patients revealed higher self-reported impulsivity and its cortical structural correlation when compared to the other SUD groups (Muller et al.).

Altogether, it has become increasingly clear that biopsychosocial factors (e.g., sex, biological predisposition, sensation seeking, adverse childhood experiences, socioeconomic vulnerability, availability, etc.) are intrinsically associated not only with the first use but with the maintenance of PSU and its consequences on cognitive dysfunction in the context of SUD. Therefore, this editorial suggests that SUD is part of a dynamic and complex system that the research field should embrace, precisely accounting for PSU patterns that, in interaction with biopsychosocial factors, might lead to cognitive dysfunction. By doing so, researchers might be able to better the individual trajectory of SUD, ultimately improving specific treatment and public policy strategies to minimize the devastating effects of this condition.

## Author Contributions

BK-S and ST wrote the first draft and final version of the manuscript. TV and RG-O contributed by providing theoretical suggestions on the topic and improving the writing process. All authors have read and approved the final article.

## Conflict of Interest

The authors declare that the research was conducted in the absence of any commercial or financial relationships that could be construed as a potential conflict of interest.

## Publisher's Note

All claims expressed in this article are solely those of the authors and do not necessarily represent those of their affiliated organizations, or those of the publisher, the editors and the reviewers. Any product that may be evaluated in this article, or claim that may be made by its manufacturer, is not guaranteed or endorsed by the publisher.
